# XUV double-pulses with femtosecond to 650 ps separation from a multilayer-mirror-based split-and-delay unit at FLASH

**DOI:** 10.1107/S1600577518006094

**Published:** 2018-08-03

**Authors:** Mario Sauppe, Dimitrios Rompotis, Benjamin Erk, Sadia Bari, Tobias Bischoff, Rebecca Boll, Cédric Bomme, Christoph Bostedt, Simon Dörner, Stefan Düsterer, Torsten Feigl, Leonie Flückiger, Tais Gorkhover, Katharina Kolatzki, Bruno Langbehn, Nils Monserud, Erland Müller, Jan P. Müller, Christopher Passow, Daniel Ramm, Daniel Rolles, Kaja Schubert, Lucas Schwob, Björn Senfftleben, Rolf Treusch, Anatoli Ulmer, Holger Weigelt, Jannis Zimbalski, Julian Zimmermann, Thomas Möller, Daniela Rupp

**Affiliations:** a Institut für Optik und Atomare Physik, Technische Universität Berlin, Hardenbergstraße 36, 10623 Berlin, Germany; b Deutsches Elektronen-Synchrotron DESY, Notkestraße 85, 22607 Hamburg, Germany; c European XFEL GmbH, Holzkoppel 4, 22869 Schenefeld, Germany; d Chemical Sciences and Engineering Division, Argonne National Laboratory, 9700 South Cass Avenue, Lemont, IL 60439, USA; e Department of Physics and Astronomy, Northwestern University, 2145 Sheridan Road, Evanston, IL 60208, USA; f optiX fab GmbH, Hans-Knöll-Straße 6, 07745 Jena, Germany; g ARC Centre of Advanced Molecular Imaging, Department of Chemistry and Physics, La Trobe University, Melbourne 3086, Australia; h Stanford PULSE Institute, SLAC National Laboratory, Menlo Park, CA, USA; i Max-Born-Institut für Nichtlineare Optik und Kurzzeitspektroskopie, Max-Born-Straße 2A, 12489 Berlin, Germany; j J. R. Macdonald Laboratory, Department of Physics, Kansas State University, Manhattan, KS 66506, USA

**Keywords:** split-and-delay unit, free-electron laser, pump–probe, multilayer mirror, temporal and spatial overlap

## Abstract

Extreme ultraviolet (XUV) and X-ray free-electron lasers enable new scientific opportunities. Their ultra-intense coherent femtosecond pulses give unprecedented access to the structure of undepositable nanoscale objects and to transient states of highly excited matter. In order to probe the ultrafast complex light-induced dynamics on the relevant time scales, the multi-purpose end-station CAMP at the free-electron laser FLASH has been complemented by the novel multilayer-mirror-based split-and-delay unit DESC (DElay Stage for CAMP) for time-resolved experiments. XUV double-pulses with delays adjustable from zero femtoseconds up to 650 picoseconds are generated by reflecting under near-normal incidence, exceeding the time range accessible with existing XUV split-and-delay units. Procedures to establish temporal and spatial overlap of the two pulses in CAMP are presented, with emphasis on the optimization of the spatial overlap at long time-delays *via* time-dependent features, for example in ion spectra of atomic clusters.

## Abstract 2

In order to probe the complex dynamics of light–matter interaction on all relevant time scales, a multilayer-mirror-based long-range split-and-delay unit was installed as a part of the CAMP end-station at the FLASH free-electron laser in Hamburg.

## 1. Introduction   

Ultra-bright extreme ultraviolet (XUV) and X-ray pulses of free-electron lasers (FELs) efficiently ionize atoms (Sorokin *et al.*, 2007 ▸) and turn condensed matter into highly excited plasma states if focused to a small spot (Wabnitz *et al.*, 2002 ▸; Chapman *et al.*, 2007 ▸). However, thanks to the short pulse duration, it is often possible to outrun the structural sample destruction and take snapshots of nanoscale matter *via* coherent diffractive imaging (CDI) (Chapman *et al.*, 2006 ▸). Furthermore, using pump–probe schemes, the ultrashort pulses allow resolving transient phenomena and dynamics in atoms, molecules and condensed matter (Radcliffe *et al.*, 2007 ▸; Frühling *et al.*, 2009 ▸; Krikunova *et al.*, 2009 ▸; Meyer *et al.*, 2010 ▸; Rompotis *et al.*, 2017 ▸; Jiang *et al.*, 2010*a* ▸; Erk *et al.*, 2014 ▸; Minitti *et al.*, 2015 ▸; Zastrau *et al.*, 2014 ▸; Vinko *et al.*, 2015 ▸; Gorkhover *et al.*, 2016 ▸; Flückiger *et al.*, 2016 ▸; for reviews see also Bostedt *et al.*, 2009 ▸, 2016 ▸; Feldhaus *et al.*, 2013 ▸; Rudenko & Rolles, 2015 ▸; Seddon *et al.*, 2017 ▸).

Of fundamental interest are the FEL-induced dynamics. One important aspect is ultrafast radiation damage, which limits the achievable resolution of CDI (Quiney & Nugent, 2011 ▸; Ziaja *et al.*, 2012 ▸; see also Garman & Weik, 2017 ▸, and references therein). More general, the high power density provides a controlled way to prepare extreme plasma states and to study them with unprecedented resolution (Bostedt *et al.*, 2012 ▸; Gorkhover *et al.*, 2012 ▸; Schroedter *et al.*, 2014 ▸; Vinko *et al.*, 2015 ▸; Ferguson *et al.*, 2016 ▸). The complex and intertwined dynamics of electrons and ions proceed on various timescales from the femtosecond to the nanosecond regime, as, for example, indicated by studies on atomic clusters (Ferguson *et al.*, 2016 ▸; Gorkhover *et al.*, 2016 ▸; Schütte *et al.*, 2015 ▸; Flückiger *et al.*, 2016 ▸). In pump–probe experiments using an optical laser, time delays up to the nanosecond regime are conceptually easier to realize. So far, existing short-wavelength split-and-delay units (SDUs) only reach pulse separations of up to a few picoseconds (Roling & Zacharias, 2016 ▸).

The CAMP instrument (Strüder *et al.*, 2010 ▸), which has been very successful in the first few years at the X-ray FEL LCLS (Bostedt *et al.*, 2016 ▸), was recently installed as a permanent end-station at beamline BL1 (Erk *et al.*, 2018 ▸) of the FLASH FEL in Hamburg (Ackermann *et al.*, 2007 ▸; Feldhaus, 2010 ▸). In order to investigate the complex FEL-induced dynamics on femtosecond to nanosecond time-scales with a second FEL pulse, the split-and-delay unit DESC was designed and integrated into the BL1 beamline in front of the CAMP end-station. With a maximum time-delay of 654 ps (maximum negative time-delay: 29 ps), this SDU enables XUV-pump–XUV-probe experiments in a, so far, unexplored time range. At the same time a (sub-)femtosecond precision also allows for the investigation of faster processes.

This paper is organized as follows. The basic concept of the split-and-delay unit is described in §2. §3 covers the key components of the setup and their implementation in the CAMP end-station at FLASH. In §4, wavelength tuning and alignment procedures required after an exchange of the mirrors are explained. §5 introduces procedures for establishing the temporal overlap (‘time zero’) in time-resolved experiments and to correct for misalignment of the spatial overlap of pump- and probe-foci when the delay is changed. As an example, time-resolved ion spectra of atomic clusters are used to illustrate the optimization procedure for long time-delays.

## 2. Concept   

### 2.1. Basic design   

Recently, the FLASH beamline BL1 was upgraded and equipped with a permanent end-station, comprising the experimental chamber CAMP (CFEL-ASG Multi Purpose chamber) (Strüder *et al.*, 2010 ▸; Erk *et al.*, 2018 ▸), a microfocusing Kirkpatrick–Baez (KB) mirror-pair (Erk *et al.*, 2018 ▸), and the split-and-delay unit DESC, which is presented in this paper.

The design of DESC was motivated by the need to cover a time-delay from femtoseconds to hundreds of picoseconds. In the XUV and soft X-ray regime, grazing-incidence angles are usually chosen for SDUs that yield a high reflectivity for a wide range of photon energies (Sorgenfrei *et al.*, 2010 ▸; Wöstmann *et al.*, 2013 ▸; Roling *et al.*, 2017 ▸); for a comprehensive review of short-wavelength SDUs, see Roling & Zacharias (2016 ▸). Grazing-incidence geometries require very long mirrors, and the achievable time-delays are commonly restricted to the order of 10 ps due to their lever arms of a few meters (Roling & Zacharias, 2016 ▸). Hence, to reach the required path difference on the order of 20 cm, multilayer mirrors and an almost normal-incidence angle were chosen.

On the other hand, a (sub-)femtosecond precision was desired, in order to enable experiments in a special operation mode, where the multi-spike pulse structure of the SASE-FEL FLASH (Self-Amplified Spontaneous Emission) (Düsterer *et al.*, 2014 ▸) is reduced to a single spike of below 10 fs duration (Rönsch-Schulenburg *et al.*, 2014 ▸). In addition, a better time resolution than given by the pulse duration was demonstrated with longer SASE pulses by making use of their spike-limited coherence length (Jiang *et al.*, 2010*b* ▸; Meyer *et al.*, 2012 ▸; Fung *et al.*, 2016 ▸).

To account for both short and long delay ranges, the movable (delay-)mirror of DESC is mounted on a combination of two linear stages for nanometer precision and decimeter range, respectively.

As a trade-off for reaching long delays, the use of multilayer mirrors restricts experiments to a single photon energy, and the reflectivity of a single multilayer mirror is usually lower than that of a grazing-incidence mirror. However, with the currently installed highly reflective multilayer mirrors optimized for the wavelength of 13.5 nm (mirror characteristics further described in §3.5) and by using a two-reflection beam path, a total transmission of 41% is achieved (the reflectivity of multilayer mirrors optimized for other wavelengths may be lower). Combined with the KB focusing optics of the BL1 beamline (Erk *et al.*, 2018 ▸), the achievable peak intensity in the interaction region is above 5 × 10^14^ W cm^−2^ for a single DESC arm and thus allows for the study of multiphoton processes (Sorokin *et al.*, 2007 ▸).

### 2.2. Beam path   

**Figure 1:**
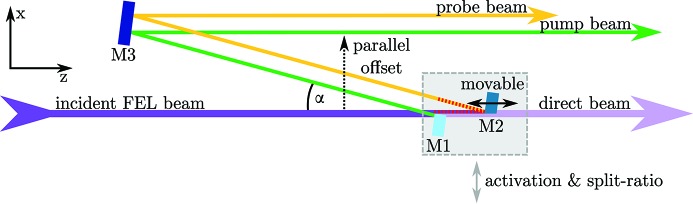
Schematic beam path of DESC (top view). For better visibility, the angle α between the incoming and reflected beam is exaggerated (in reality α = 0.7° and the distance between mirrors M1 and M3 is 1166 mm). DESC is activated by jointly moving M1 and M2 (gray box) into the incident FEL-beam (along the *x*-axis), which is then split into two parts. Mirror M1 is fixed along the *z*-axis, M2 is installed on two linear stages (short- and long-range) for delaying one part of the beam (yellow) with respect to the other (green). The red dashed lines illustrate the optical path difference. Both beams are reflected in the downstream direction (towards the KB optics and experimental chamber) by a third multilayer mirror M3, with a parallel (*x*-)offset (indicated by the dashed-dotted arrow) with respect to the incident direct FEL-beam (SDU not active). All mirrors can be adjusted in pitch and tilt (*cf*. §3.4).

The incident FEL wavefront is split by two mirrors and reflected onto a third mirror, which guides both beams downstream to the CAMP instrument (see Fig. 1 ▸). A difference in optical path length, and, thus, a time-delay between the two pulses can be achieved by moving one of the split-mirrors along the axis of the incident FEL-beam. This near-normal-incidence geometry based on multilayer mirrors allows for a compact beam path design.

A reasonable high transmission is achieved with DESC since only two reflections are needed; usually four or more reflections have to be used for grazing-incidence SDUs (Roling & Zacharias, 2016 ▸). We note that another long-delay approach *via* multilayer mirrors is based on four 45° reflections (Roling & Zacharias, 2016 ▸).

**Figure 2:**
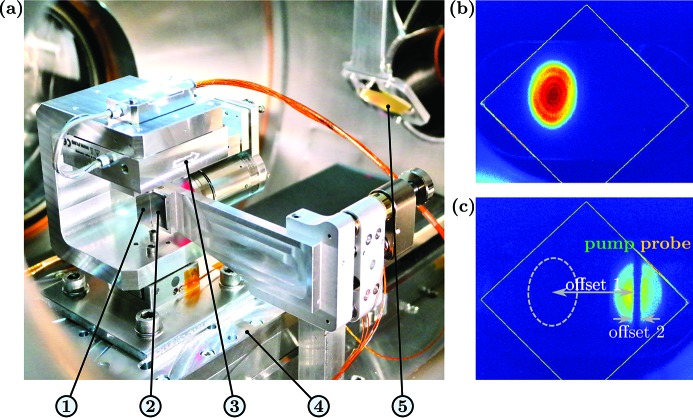
(*a*) Photograph of the split-and-delay unit (shown at zero delay): (1) Multilayer mirror M2 which can be moved with a (3) short-range or (4) long-range linear stage with respect to the (2) fixed multilayer mirror M1. (5) Fluorescent Ce:YAG screen, which can be introduced into the beam path for monitoring the beam(s). (*b* and *c*) Camera view of the FEL-beam(s) on the Ce:YAG screen: (*b*) intensity profile without SDU, (*c*) intensity profile at active SDU and 50%/50% split-ratio at the full delay. Note that a lateral offset of the split-beam with respect to the incoming FEL results from the *z*-shape of the DESC beam path (offset 1). In addition, the finite incident angle α leads to a time-delay-dependent gap in between pump- and probe-beam (offset 2). The ellipsoidal shape of the beam profiles is caused by a 45° angle of incidence on the screen. The ring structures overlaying the intensity profiles originate from beamline-apertures in the FEL tunnel. The bright oblique lines indicate the edges of the screen (edge length = 25 mm).

DESC is placed before the KB focusing optics and is activated by moving the split-mirrors into the FEL-beam [motorized split-and-delay unit in Fig. 2(*a*) ▸]. The mirrors M1 and M2 split the incident wavefront into two horizontally separated parts. Split-ratios other than 50%/50% [as shown in Fig. 2(*c*) ▸] can be adjusted flexibly by changing the lateral position of the split-and-delay setup. Both beam parts are reflected under almost normal incidence on mirror M3, which directs the beams into the experimental chamber with a parallel offset of ∼15 mm with respect to the incident FEL-beam [offset 1, center to center, see Fig. 2(*c*) ▸]. This parallel offset is compensated by moving the chamber for the KB optics and the CAMP chamber accordingly; both are installed on rails and on a fully motorized frame, respectively.

With this approach of wavefront splitting, a small percentage of photons is lost in the gap between M1 and M2 since a certain distance of the order of 200 µm is needed to avoid a collision when reaching time-zero. Note that changing the time-delay is accompanied by an offset of the probe beam with respect to the pump beam [offset 2, see Fig. 2(*c*) ▸, compare also the beam path in Fig. 1 ▸]. In other words, the large gap between pump- and probe-beam up to 1 mm, observed at longer delays on the Ce:YAG screen [*cf*. Fig. 2(*c*) ▸], is not associated with further photon loss.

In order to transport the split-beam into the CAMP interaction region without clipping the split-beam by the geometric acceptance of the KB optics of ∼10 mm, its full diameter (pump-beam and probe-beam, plus gap) cannot exceed this value. This is realized by the very small design angle of α = 0.7° between incoming and reflected beam.

### 2.3. Time-delay   

The time-delay between pump- and probe-pulse is achieved by moving the mirror M2 parallel to the incident FEL-beam axis *via* the combination of a short-range and a long-range linear stage, as further detailed in §3.4 and Table 2. The time-delay is calculated *via* the formula
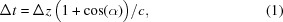
with 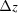 = 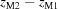 being the distance between the positions of the mirrors M1 and M2 along the *z*-axis, 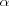 = 0.7° being the full angle between incident FEL-beam and reflected beam (*cf*. Fig. 1 ▸) and *c* the speed of light in a vacuum.

Temporal overlap by means of zero time-delay (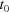) occurs when both mirrors are at the same position along the *z*-axis, *i.e.* at 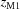 = 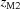 [*cf*. Fig. 2(*a*) ▸]. As explained in §5.1, in a recent time-resolved experiment, the temporal overlap of pump- and probe-pulses was identified at a position of the long-range stage of 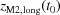 = 4.38 mm (full range 102.49 mm), and of the short-range stage at 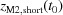 = 125.65 µm [full range 250 µm; note that a position of 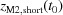 close to mid-range was chosen on purpose]. Thus 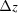 is calculated by
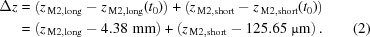
The exact values given here have to be checked and/or re­established for each experiment. A detailed description for this procedure is given in §5.1.

## 3. Implementation   

### 3.1. Vacuum apparatus   

**Figure 3:**
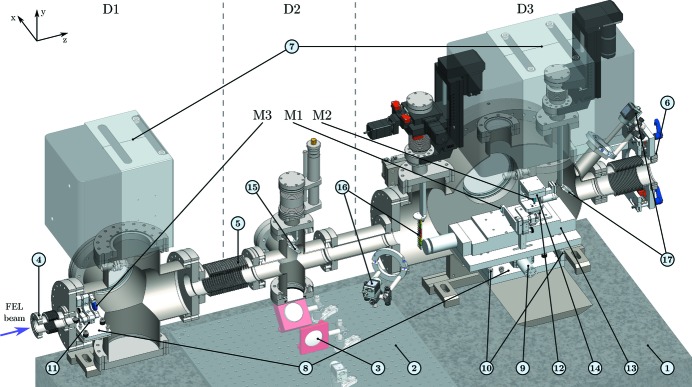
Overview of the DESC components. D1, D2, D3: vacuum chambers (upstream to downstream). M1: fixed multilayer mirror installed in an open-loop piezo actuators mirror mount. M2: delay multilayer mirror installed on a closed-loop piezo tip/tilt platform. M3: back-reflecting multilayer mirror installed in an open-loop piezo actuators mirror mount. (1) Granite stone, (2) breadboard on top of granite stone, (3) optical setup for beamline laser, (4) bellow at incident FEL-beam, (5) bellow compensating elongation during bake-out, (6) rail-guided bellow required for lateral offset split-beam in active SDU, (7) ion getter pumps, (8) breadboards on a three-point bearing (on top of chamber breadboards), (9) linear stepper motor for SDU activation, (10) guide rails supporting aluminium board on the top, (11, 12) piezo-driven small linear stages, (13) stepper-motor-controlled long-range delay stage (shown at full delay), (14) piezo-driven short-range delay stage, (15) optical beamsplitter for alignment with the beamline laser, (16) set of filters for attenuation of pump- and probe-beam with a camera for alignment, (17) Ce:YAG and diffusing screen (for beamline laser) with a camera for beam monitoring.

As shown in Fig. 3 ▸, the SDU is placed in a vacuum apparatus consisting of three interconnected chambers (D1, D2, D3). From upstream to downstream, the incident FEL-beam first passes chamber D1 housing M3 and chamber D2, which contains an optical beamsplitter for the initial alignment procedure (*cf*. §4.1). Finally, in chamber D3, the FEL-beam is split and delayed by the wavefront split-mirrors M1 and M2, and sent back upstream.

Chamber D3 is based on a DN 400 CF tube, providing sufficient space for comfortable installing and maintaining the interior as well as for exchanging mirrors M1/2 through DN 400 CF door openings. Also, the third mirror M3 in D1 can be easily accessed through DN 150 CF flanges. The whole apparatus measures 1.8 m along the *z*-direction (beam axis) and 0.5 m in the *x*-direction, thus being relatively compact considering the achievable long time-delays in the short-wavelength regime.

The SDU is operated at a base pressure of 10^−9^ mbar, sufficient for a low contamination of the mirrors and consequently a long lifetime at high reflectivity. For this reason, all installed materials and motors were chosen to be ultra-high-vacuum compatible. The chamber is opened (*e.g.* for mirror change) using a mobile flow box in order to keep dust contamination of the setup as small as possible. Pumping down to the 10^−6^ mbar range is achieved with a turbomolecular pump, typically after ∼36 h. Then, two ion getter pumps (410 l s^−1^ overall) are started, and the turbomolecular pump is switched off and disconnected, isolated by a metal sealed valve. Ion pumps were chosen to avoid mechanical vibrations and for their almost maintenance-free operation. Reaching the 10^−9^ mbar range takes 20 to 30 days mainly affected by out-gassing of motors and cables. Bake-out of the apparatus can accelerate the pumping process, reaching the 10^−9^ mbar range after ∼10 days (maximum 80°C, limited by in-vacuum motors). A flexible bellow between D1 and D2 accounts for elongation of the apparatus during the bake-out process with respect to the granite base (*cf*. §3.3). Temporal and spatial overlap might be altered by the bake-out and have to be rechecked (*cf*. §5.1, §5.2).

It is important to note that pumping down to 1 mbar and venting to 1 bar has to be done very slowly (within at least 1 h) to avoid destruction of the installed ultrathin filter foils (*cf*. following §3.2).

### 3.2. Beam monitoring and filters   

Precise control of the beam path is essential for an accurate alignment. For monitoring the direct beam as well as the double-pulses, a fluorescent Ce:YAG screen (stacked on a diffusion screen for the optical beamline laser) is fixed on a motorized *z*-manipulator and can be introduced into the path of the exiting beams [*cf*. Fig. 2 ▸ and (17) in Fig. 3 ▸]. For initial alignment purposes with the optical beamline laser, a glass beamsplitter may also be introduced into the beam path halfway (chamber D2) between M1/2 and M3 [*cf*. Fig. 3 ▸, (15)], as further detailed in §4.1.

A set of zirconium filter foils [Fig. 3 ▸, (16)] offers the possibility to vary the intensity ratios of the pump- and probe-pulses at a geometrical split-ratio of 50%/50% [as in Fig. 2(*c*) ▸]. Currently, Zr filters with a thickness of 300 nm are installed on a motorized *xyz*-manipulator, attenuating the beam at 13.5 nm wavelength down to one-third (Henke *et al.*, 1993 ▸). In addition, Zr filters with a thickness of 650 nm [10% transmission (Henke *et al.*, 1993 ▸)] are available but have to replace the 300 nm foils. The filter holder also includes blockers for separately blocking the pump- or probe-beam.

In case of an exchange of the multilayer mirrors for other wavelengths (*cf*. §4.4), the filters can be adapted as well. Transmission curves can be calculated online using the CXRO X-ray database, based on Henke *et al.* (1993 ▸).

### 3.3. Beamline integration   

DESC is placed upstream of the CAMP experiment and the KB focusing optics. The required space of the existing beamline-infrastructure (cooling water, gas lines, cable tray) prevented a direct mounting of the SDU to the floor. Hence, a bridging aluminium frame was chosen, supporting a 650 kg granite stone, that reduces vibrations from the surrounding experimental hall, with the chambers fixed on top.

As described in §2.2, the active mode of DESC is accompanied by the parallel offset of the split-beam in the horizontal plane [offset 1 (*x*-axis), *cf*. Figs. 1 ▸ and 2(*c*) ▸]. To allow for lateral displacement of all downstream beamline components [*i.e.* connecting parts to KB optics, chamber with KB optics, CAMP chamber (Erk *et al.*, 2018 ▸)], a rail-guided DN 63 CF bellow is mounted at the exit of DESC.

A separation of the vacuum system by two gate valves, directly upstream and downstream of DESC, enables independent vacuum operation of the different beamline sections, *e.g.* for mirror exchange.

### 3.4. In-vacuum motors   

The whole SDU is controlled by eight in-vacuum motors, which can be classified into two categories (see Table 1 ▸): (*a*) those needed for the operation of DESC, *i.e.* before or during each experiment, and (*b*) those necessary for alignment procedures, *e.g.* after an exchange of mirrors.

DESC is activated by moving mirrors M1 and M2 horizontally into the incident FEL-beam [*cf*. Fig. 2(*a*) ▸] with a stepper-motor-controlled linear stage. For this purpose, the split-and-delay setup in chamber D3 is situated on a rail-guided aluminium support plate. The beam split-ratio can be monitored on the fluorescent Ce:YAG screen at the exit of DESC (*cf*. Fig. 2 ▸).

Time-delays between pump- and probe-beam are adjusted by a short-range and a long-range linear stage. The short-range delay stage is mounted on top of the long-range delay stage. Both stages move mirror M2 along the incident FEL-beam. The piezo-operated short-range delay stage allows a bi-directional movement in 14 attosecond (as) time-steps with a repeatability of 14 as. However, fluctuations of roughly ±200 as (encoder read-out) due to mechanical vibrations of the SDU limit the absolute temporal resolution. While the range of the short-range delay stage is ±830 fs, the long-range delay stage allows up to 654 ps time-delay with a step size of 7 fs, a uni-directional repeatability of 2 fs and a bi-directional repeatability of 7 fs.

The short-range delay stage uses an encoder to determine the position, while the position for the long-range delay stage is calculated by the steps of the motor. The main specifications of the delay stages are listed in Table 2 ▸.

Inevitably, adjusting time-delay induces a loss of spatial overlap of the foci due to the pitch and yaw movement of the delay stages connected to their finite mechanical precision, as further discussed in §5.2. For quick realignment, mirror M2 was equipped with a fast adjustable closed-loop mirror mount. Here, a piezo-driven tip/tilt platform was chosen, allowing for 0.05 µrad resolution and 0.15 µrad repeatability. Fluctuations of ±0.2 µrad observed during operation probably originate from mechanical vibrations.

Pitch and tilt movement of mirrors M1 and M3 (not needed during normal operation) are carried out by open-loop long-term stable piezo actuators. The gap between mirrors M1 and M2 (*x*-axis) is minimized with the help of a small linear piezo-driven stage, which moves M2. Because of the small distance between mirror M3 and the incident FEL-beam, M3 is mounted on a small linear piezo-driven stage along the *x*-axis. If larger FEL diameters than usual were required in inactive SDU mode, this stage would allow moving M3 out of the beam.

### 3.5. Mirrors   

**Figure 4:**
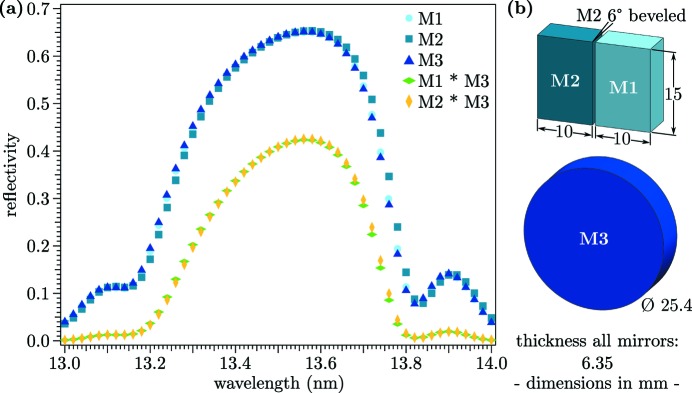
(*a*) Reflectivity *versus* photon wavelength of the currently installed multilayer mirrors. Blue: manufacturer data (measured by PTB@BESSY II); yellow and green: total reflectivity of pump-beam path and probe-beam path, calculated by M1 * M3 and M2 * M3, respectively. Here a reflectivity of 41% is reached at 13.5 nm. At a geometrical split-ratio of 50%/50%, each beam transmits ∼20% of the originally incident photons. (*b*) Geometrical dimensions of the mirrors.

Photon energies at FLASH1 may be tuned from 24 eV to 295 eV in the fundamental (Schreiber & Faatz, 2015 ▸), a range that is fully covered by suitable multilayer materials (Bourassin-Bouchet *et al.*, 2015 ▸). Currently, a set of flat multilayer mirrors with a design wavelength of 13.5 nm is installed in the SDU. The multilayers consist of 50 periods of molybdenum/silicon on a fused silica substrate (surface flatness λ/20), with the thickness of every single period of 6.900 ± 0.005 nm (Feigl *et al.*, 2006 ▸). A maximum reflectivity of 64% at a wavelength of 13.5 nm (∼91.8 eV) for each mirror is achieved. The transmission through DESC, after passing M1 (or M2) and M3, is calculated *via* convolution of the particular reflectivities, to be 41%, as visualized in Fig. 4(*a*) ▸. Measured transmissions have been observed to be lower sometimes (*cf*. §4.2), maybe depending on FEL tuning conditions.

The mirrors are exchangeable for other desired wavelengths; replacement mirrors would have to be user provided. However, the costs are comparatively low because of the small mirror dimensions, resulting from the near-normal-incidence geometry. The mirror sizes are specified in Fig. 4(*b*) ▸.

The facing planes of M1 and M2 were polished for sharp edges, resulting in an unimpaired splitting of the incident wavefront. Since the distance between the edges of M1 and M2 is very small (of the order of 200 µm), the inner plane of M2 was beveled in order to avoid a collision when M2 passes M1 towards negative time-delays.

## 4. Alignment procedures and wavelength matching   

### 4.1. Initial alignment   

During the initial setup of DESC, after adjustment of the chambers on the granite stone, all mirrors and motors were leveled with a three-point bearing on their breadboards and fixed afterwards. A rough beam alignment on beamline laser was realized using large-angle mirror mounts. Note that since the range of the tip/tilt platform for M2 is limited to ±1 mrad, M2 is additionally installed in a manual mirror mount with a large angular range, which can be adjusted when the chamber is opened.

Theoretically, an ideal beam alignment of DESC is reached when the incident angles on the KB mirrors with and without DESC are identical. Then, the focus quality in the CAMP chamber is optimal. Such an ideal alignment can be achieved *via* two steps. First, a parallel path of the outgoing split beams with respect to the direct FEL-beam is established using the optical beamline alignment laser, which is discussed below in this section. Second, the slight change of the angles of the KB chamber itself, induced by its lateral motion, is compensated *via* a direct optimization of the FEL-beam angles by tuning on focal power density in the CAMP chamber, as described in §4.3.

To achieve the first step, *i.e.* a parallel alignment of the outgoing split beams with respect to the incident beam, an out-of-vacuum optical setup is used, as depicted in Fig. 3 ▸, (2, 3). An optical beamsplitter mounted on a linear manipulator in chamber D2 is moved into the beam path of the beamline laser [Fig. 3 ▸, (15)]. With an incident angle of 45° and a 30%/70% intensity splitting ratio (reflected/transmitted), both the incident beam and the split-beam are coupled out side-by-side through a viewport towards the out-of-vacuum breadboard. There, the setup essentially consists of a lens and a camera within the focal plane of the lens. By spatially overlapping all three focal spots (incident beam, split beams), a parallel path of the split-beam relative to the incident beam is ensured.

### 4.2. Wavelength tuning   

An exact wavelength tuning of FLASH prior to any experiment using DESC is crucial, since a deviation of only 0.3 nm from the ideal wavelength would cause a drastic transmission loss [*cf*. Fig. 4(*a*) ▸]. Matching the wavelength to the mirrors’ reflectivity may be performed by an absolute measurement with the plane-grating monochromator at the PG beamlines (Martins *et al.*, 2006 ▸; Gerasimova *et al.*, 2011 ▸), or by tuning directly on an optimized transmission through DESC, as briefly described in the following.

The intensity profiles of the direct beam and the transmitted beam can be observed on the same Ce:YAG screen mounted on the beam monitor at the exit of DESC (*cf*. §3.2). The ratio of the transmitted beam intensity and the direct beam intensity is determined [see, for example, different intensities on the Ce:YAG in Figs. 2(*b*) and 2(*c*) ▸] and tuned for maximum values. As tested in several experiments, tuning on the highest intensity ratios can be used to match the wavelength to the reflectivity window of the multilayer mirrors of DESC.

### 4.3. Focus optimization   

In addition to a residual misalignment of the beams through DESC after the steps described in §4.1, the lateral shifting of the chamber with the KB optics into active SDU mode also slightly changes the angles of incidence compared with the position of inactive SDU mode, caused by inevitable pitch and yaw errors of its rails. However, having the mirror angles optimized once, we found the change in pitch and yaw angles of the KB optics between the modes ‘DESC inactive’ and ‘DESC active’ to be reproducible when the KB chamber was moved between precise limit stoppers. Therefore, one can automatically correct these angular offsets when switching between modes.

The focal waist of the KB optics is extremely sensitive to the incident angles of the beam. Hence, we use a power-density-dependent signal from the focal region in the center of the CAMP chamber for an exact alignment of the SDU mirrors in terms of smallest foci. The focus quality can, for example, be characterized by nonlinear ionization processes, *e.g.* of xenon gas, and optimizing the creation of highest ion charge states (*cf*. Erk *et al.*, 2018 ▸).

To rule out any double-pulse effects during focus optimization of DESC, pump- and probe-beams are aligned separately. Because the delay mirror M2 is mounted on a fast and reproducible closed-loop mirror mount (*cf*. Table 1 ▸), the probe-beam path is aligned first by reflecting 100% of the incident FEL-beam on M2 and adjusting M2 and M3 iteratively. Subsequently, the pump-beam path can be easily aligned. The full FEL-beam is now reflected by M1 and the M2 alignment is retraced *via* digital markings on the DESC beam monitor [Fig. 3 ▸, (17)] and a fluorescence screen in the focus of CAMP. As a final step, the pitch and tilt angles of M1 are fine-tuned, *e.g.* by optimizing on high xenon charge states.

**Figure 5:**
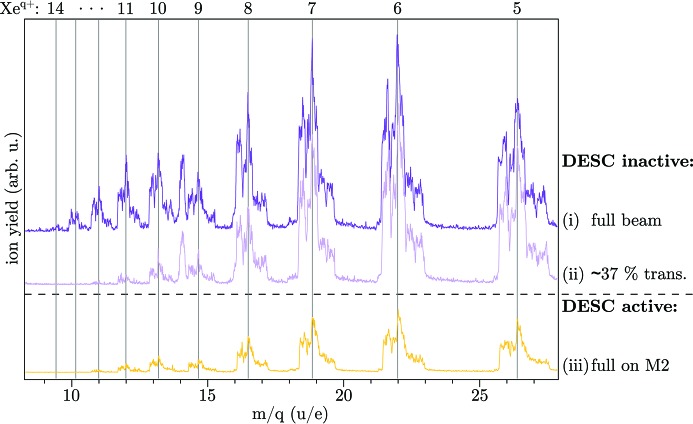
Ion spectra from xenon gas, zoomed in on high charge states. Comparison for (i) DESC inactive, (ii) attenuated intensities at inactive DESC with filter foil, imitating the transmission of DESC and (iii) DESC active. The time-of-flight measurements were averaged and converted to mass-over-charge ratio. Without DESC, the highest observed charge state was reduced from Xe^14+^ at full beam to Xe^12+^ using a 200 nm niobium foil (transmission ∼37%). After iteratively optimizing all mirrors of DESC for highest charge states, 12-fold ionized xenon was also observed, indicating a good alignment of DESC. We note that the total lower ion yield at (iii) is induced by slightly changed positions of the retractable time-of-flight spectrometer, which is not relevant for the appearance of the high charge states. Besides Xe^*q*+^, N^+^ at *m*/*q* = 14 from residual gas can also be observed in the ‘DESC inactive’ measurements, which was present only at the beginning of the experiment.

For a quantitative comparison of the KB focus qualities with and without DESC, beamline filter foils can be used to attenuate the direct beam (DESC inactive) in order to match the reduced transmission through the SDU. Fig. 5 ▸ illustrates the outcome of the focus optimization procedure in a commissioning beam time in summer 2016 after mechanical changes in DESC. Here, the FEL was tuned to 100 µJ pulse energy at 13.5 nm wavelength [single bunch, not including beamline transmission, *cf*. Erk *et al.* (2018 ▸)], and xenon gas with a partial pressure of ∼1 × 10^−6^ mbar was introduced into the CAMP chamber. In active DESC mode, a maximum xenon charge state *q* = 12 was found (full beam on probe-arm). The measurement with reduced transmission in inactive DESC mode [∼37%, using a 200 nm niobium foil (Henke *et al.*, 1993 ▸)] resulted in the same charge state, indicating a good alignment of DESC.

### 4.4. Mirror exchange   

All mirrors are accessible through large flanges (*cf*. §3.1). This allows the installation of multilayer mirrors optimized for wavelengths other than 13.5 nm, if required in future experiments. As mentioned, to keep the beamline and the interior of DESC dust free, a portable flow box should be used. By using the alignment of the installed mirrors, the successive exchange of one mirror after the other will allow for a quick alignment restoration. For instance, the focus positions on a screen in the CAMP chamber provide a long lever and thereby a precise marking. The alignment should be checked and may be optimized after all mechanical changes in DESC, and procedures to establish temporal and spatial overlap have to be repeated (*cf*. §5).

## 5. Temporal and spatial overlap   

### 5.1. Temporal overlap   

Precise knowledge of the temporal relation between pump- and probe-pulses is necessary for any time-resolved experiments. Thus, establishing a ‘zero delay’ time reference is essential. In particular after any mechanical change in the DESC setup such as the exchange of mirrors, reestablishing of the temporal overlap between pump- and probe-pulses is required. In addition, it is recommended to confirm ‘time zero’ as a check-up procedure before every experiment.

To this aim, the two split beams can be overlapped on a fluorescence screen, providing access to a spatial interference pattern in case of temporal overlap (Mitzner *et al.*, 2008 ▸). As the procedure does not rely on a focused beam, it is not necessary to correct for pitch and yaw motion of the mirror M2 (*cf*. §5.2) while scanning time-delay for temporal overlap. However, considering the femtosecond pulse duration and the particularly short longitudinal coherence time of a SASE FEL such as FLASH (Mitzner *et al.*, 2008 ▸; Schlotter *et al.*, 2010 ▸; Roling *et al.*, 2011 ▸), the determination of temporal overlap starting from an arbitrary position can become a tedious and time-consuming procedure. As the fringe pattern is only visible within the delay time interval corresponding to the FEL pulse coherence time, typically in the sub-10 fs range, a small step size within a fairly large scanning window is required. When found, maximizing the fringe pattern visibility ensures that the two partial pulses arrive in the common focal region in a sub-10 fs interval, which is typically shorter than the FEL pulse duration.

**Figure 6:**
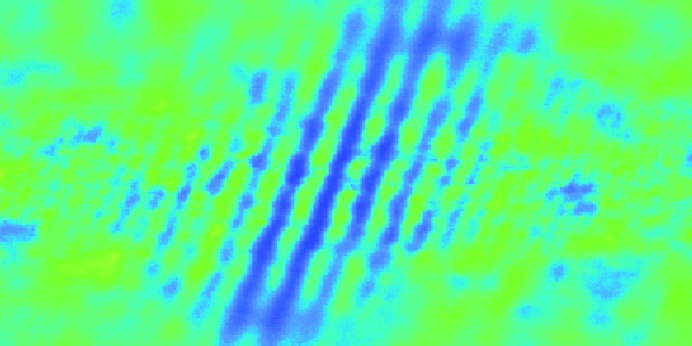
Interference pattern at maximum visibility of partially overlapping pump- and probe-beams on a fluorescent screen ∼80 cm after the focus in CAMP. The fringes appear at temporal overlap (zero time-delay) and disappear at a few femtoseconds time-delay, consistent with the coherence time of the FLASH FEL at 13.5 nm (see text). Note that in order to overlap both beams on the screen, focal overlap had to be abandoned during this measurement.

In a recent experiment, zero time-delay at 13.5 nm wavelength was identified at a short-range delay stage position of 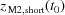 = 125.65 µm while the long-range delay stage was at position 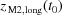 = 4.38 mm. An interference pattern, shown in Fig. 6 ▸, was clearly visible on a screen ∼80 cm behind the focus of CAMP, confirming temporal overlap, after it had been initially determined by ion signal over the course of the respective experiment. The pattern was observable within a position range of the short-range delay stage of less than 1.5 µm, corresponding to a sub-10 fs longitudinal coherence length of the FLASH FEL, being in good agreement with a previous finding at this wavelength (Roling *et al.*, 2011 ▸).

Note that under consideration of the limited bi-directional repeatability of ±1 µm of the long-range delay stage (*cf*. Table 2 ▸), the value of 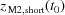 may vary if the long-range stage is moved.

### 5.2. Spatial overlap of pump- and probe-foci   

Pump–probe experiments usually require high intensities, and thus the use of small foci. When changing time-delay, the spatial overlap of the two foci of the pump- and probe-beam is often lost due to the pitch and yaw movements of the delay stages resulting from finite fabrication precision. Caused by the lateral offset 2 of the probe-beam [*cf*. Fig. 2(*c*) ▸], the virtual FEL source point of the diverging FEL beam is also laterally offset (Campi *et al.*, 2016 ▸). Taking the magnification of the KB optics into account, this translates into a lateral offset of the probe-focus of up to 10 µm at maximum time-delay. Therefore, keeping the spatial overlap while changing the time-delay is an important, though challenging, task.

Generally, all correction motions in DESC are carried out by the pitching and tilting mirror M2 using the closed-loop actuator. The displacement of the angles from the two delay stages is not fully reproducible and changes systematically in succession after mechanical changes (*e.g.* exchanging mirrors). Thus, look-up tables with correction values for pitch and yaw angles are measured on a regular basis and can be used as a first-order correction. Nevertheless, the values have to be checked and adapted for each experiment.

At the CAMP end-station, several tools are available for creating look-up tables and fine-tuning of the overlap. A rough overlap can be established by direct monitoring of the foci with the help of a Ce:YAG screen in combination with a microscope and placing the probe-beam on top of the pump-beam. It should be ensured that the Ce:YAG screen is in the focal plane by scanning it along the beam axis. However, this method is limited by the resolution of the microscope and saturation of the fluorescent signal.

Alternatively, a velocity map imaging (VMI) spectrometer (Erk *et al.*, 2018 ▸), operated in spatial imaging mode and in time-of-flight mode, is a convenient tool for gas-phase experiments to overlap both foci in all three dimensions with micrometer precision (Johnsson *et al.*, 2010 ▸). Using proper voltage settings and a gas-flooded chamber, the beam profiles can be spatially imaged, allowing for a direct observation of the focus waist and enabling a comfortable way to overlap the foci along the *x*-axis. By matching the flight times of ions, it is possible to correct for offsets in height (*y*-axis, *cf*. the coordinate system in Fig. 3 ▸).

The spatial overlap achieved *via* Ce:YAG screen or VMI spectrometer should be cross-checked and further fine-tuned *via* an overlap-dependent signal from the focus, *e.g.* on ions. For short time-delays, a delay scan around temporal overlap on a highly charged gas ion signal, giving access to high-order intensity autocorrelation functions of the radiation field, should clarify the spatial overlap (Mitzner *et al.*, 2009 ▸; Moshammer *et al.*, 2011 ▸; Rompotis *et al.*, 2017 ▸). For long time-delays in the picosecond range, different mechanisms could be used. As an example, we describe an approach using gas-phase clusters in the following section.

### 5.3. Overlap optimization on ion spectra from rare-gas clusters   

Optimizing the overlap of pump- and probe-foci in the CAMP chamber at long delays requires an overlap-dependent signal that is still present at delays of several hundreds of picoseconds. Dynamics up to the picosecond and nanosecond regime have been reported in ion spectra from atomic clusters (Krikunova *et al.*, 2012 ▸; Schütte *et al.*, 2015 ▸; Flückiger *et al.*, 2016 ▸). For the commissioning of DESC and first user experiments, xenon clusters were utilized for overlap optimization, as the overlap-dependent signatures are clearly visible, very sensitive to ideal overlap, and even increase in strength for long delays.

**Figure 7:**
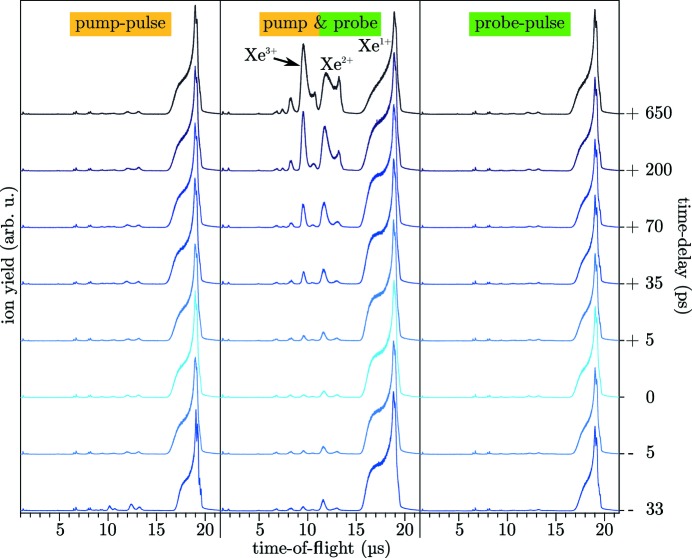
Time-of-flight ion spectra from xenon clusters with an average size of 24 nm in radius, averaged over 200 single FEL shots. The central column presents pump–probe ion spectra from −33 ps to 650 ps (the maximum delay values are not fully identical to Table 2 ▸ due to a different mirror holder setup at that time). The signal from higher charge states increases with time-delay, suitable for optimizing the spatial overlap at long time-delays. The left and right columns show corresponding spectra of pump- or probe-pulse only, each obtained with a relatively low power density of about 2.0 × 10^12^ W cm^−2^ at 13.5 nm wavelength. Note that the additionally visible charge states in the pump-only spectrum at −33 ps result from an experimental artifact (the probe-pulse was not fully blocked at this data point).

As an example, in Fig. 7 ▸ time-of-flight ion spectra of xenon clusters with ∼24 nm radius are shown. They were measured during the course of the first user experiment. The ion spectra were recorded using a look-up table for readjusting the mirror M2 that had been created several hours before under different cluster generation conditions, suggesting a satisfying reproducibility on the scale of one beam-time shift. The correction values had been determined by iteratively optimizing pitch and tilt of M2 by tuning on maximum yield of the higher charged xenon ions for all displayed delays.

The left and right columns of Fig. 7 ▸ display the spectra of the pump- or probe-pulse alone, which do not change with the applied delay. In the center column the ion spectra of double-pulse excited clusters are given, showing a striking increase of higher charge states of xenon with a longer delay between the two pulses.

The physics underlying these dynamics is well understood (Arbeiter *et al.*, 2014 ▸). While the same total amount of energy impinges on the clusters at all time-delays, the recombination following the ionization is strongly suppressed for XUV double-pulses with long delays. The reason for this suppressed recombination lies in the expansion of the cluster nanoplasma after the excitation by the first pulse (Arbeiter *et al.*, 2014 ▸). Generally, the expansion leads to a cooling of the electrons as they transfer their kinetic energy to the ions of the cluster. This cooling effect is decreased for the electrons created by the second pulse which, subsequently, are less likely to recombine.

## 6. Summary and outlook   

The multilayer-mirror-based split-and-delay unit DESC was set up as a part of the beamline BL1 at the CAMP end-station at the free-electron laser FLASH. By selecting a single wavelength with multilayer mirrors, a beam path design became feasible which allows up to 654 ps time-delay. Furthermore, (sub-)femtosecond steps enable experiments within the coherence time of FLASH. The mirrors are exchangeable for operating DESC at different fixed wavelengths.

The presented SDU creates intense XUV double-pulses reaching into a, so far, unexplored time regime. The parameters of DESC offer the possibility to disentangle fast and slower dynamics such as excitation, ionization, charge transfer and structural dynamics in highly excited non-equilibrium states of matter within a single experimental approach. For example, over the course of the experiment investigating xenon clusters (Fig. 7 ▸), also the light-induced expansion of single xenon clusters up to 650 ps was followed *via* coherent diffractive imaging as a new avenue to study XUV-induced structural changes.

In conclusion, DESC provides novel possibilities for time-resolved experiments using intense XUV pulses with characteristics complementary to other XUV split-and-delay units. It is commissioned and operational for experiments at the CAMP end-station of the FLASH free-electron laser.

## Figures and Tables

**Table 1:** Overview of the in-vacuum motors for (*a*) DESC operation and for (*b*) DESC alignment procedures after mirror exchange See text for a detailed explanation and Fig. 3 ▸ for axes definition. Abbreviations: ol = open loop; cl = closed loop (encoder feedback).

	Task	Motor	Axis	Type
(*a*)	Activation/split-ratio	Linear stage	*x*	Stepper, ol
	M2: (sub-)fs-delay	Linear stage, short-range	*z*	Piezo, cl
	M2: ps-delay	Linear stage, long-range	*z*	Stepper, ol
	M2: pitch and tilt	Tip/tilt platform	–	Piezo, cl

(*b*)	M1 and M2: gap	Linear stage	*x*	Piezo, ol
	M3: positioning	Linear stage	*x*	Piezo, ol
	M1: pitch and tilt	Mirror mount	–	Piezo, ol
	M3: pitch and tilt	Mirror mount	–	Piezo, ol

**Table 2:** Main specifications of the short-range and long-range linear delay stages All times 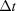 are calculated with equations (1) and (2) and rounded up to whole numbers, except for maximum positive delays which are rounded down.

	Delay stage			
	Short-range		Long-range	
Characteristic	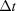	*z*	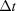	*z*
Maximum negative delay	838 fs	125.65 µm	29 ps	4.38 mm
Maximum positive delay	829 fs	124.35 µm	654 ps	98.11 mm
Step size	14 as	2 nm†	7 fs	1 µm
Uni-directional repeatability	‡	‡	2 fs	0.2 µm
Bi-directional repeatability	±14 as	±2 nm	±7 fs	±1 µm

† The encoder shows fluctuations of roughly ±30 nm (= ±200 as) caused by mechanical vibrations.

‡ Closed-loop operation (encoder), hence no uni-directional repeatability is defined.
